# Performance and Training Load Profiles in Recreational Male Trail Runners: Analyzing Their Interactions during Competitions

**DOI:** 10.3390/ijerph17238902

**Published:** 2020-11-30

**Authors:** Sérgio Matos, Filipe Manuel Clemente, Rui Silva, Joel Pereira, José María Cancela Carral

**Affiliations:** 1 Faculty of Educational Sciences and Sports Sciences, University of Vigo, 36005 Pontevedra, Spain; chemacc@uvigo.es; 2 Escola Superior Desporto e Lazer, Instituto Politécnico de Viana do Castelo, Rua Escola Industrial e Comercial de Nun’Álvares, 4900-347 Viana do Castelo, Portugal; rui.s@ipvc.pt (R.S.); joelpereira@esdl.ipvc.pt (J.P.); 3 Douro Higher Institute of Educational Sciences, 4560-708 Penafiel, Portugal; 4 Instituto de Telecomunicações, Delegação da Covilhã, 1049-001 Lisboa, Portugal; 5 Unidade de Investigação e Treino em Trabalhos em Alturas e Atividades de Ar Livre, 4960-320 Melgaço, Portugal; 6 The Research Centre in Sports Sciences, Health Sciences and Human Development, 5001-801 Vila Real, Portugal

**Keywords:** sports training, monitoring, endurance sports, trail running, periodization

## Abstract

Endurance sports like trail running constitute an extensive individual modality causing numerous physiological changes to occur in the athlete. In this sense, an adequate monitoring of training load appears to be essential to improve competition performance. The aim of this study was two-fold: (i) to analyze trail runners’ weekly load variations in the four weeks leading up to a trail running competition, and (ii) to determine the relationship between the runners’ pacing in competitions and their physical fitness and workload parameters. Twenty-five amateur male trail runners (age: 36.23 ± 8.30 years old; minimum International Trail Running Association performance index: 600) were monitored daily for the duration of a season (52 weeks). External load (distance covered, pace) and internal load (rate of perceived exertion) were measured daily. Additionally, weekly workload measures of acute:chronic workload ratio (ACWR), training monotony, and training strain were calculated. The runners were also assessed for maximal aerobic speed (MAS) every four months. No significant differences in workload measures (*p* > 0.05) were observed in the four weeks leading up to each short trail competition; however, leading up to the long trail, ultra-trail medium, and ultra-trail long/extra-long competitions, the differences in the runners’ workload measures were significant (*p* < 0.05). In the short trail, pace was found to be moderately correlated with the ACWR of total distance (r = −0.334) and with training monotony of rate of perceived exertion (RPE) (r = −0.303). In the ultra-trail, a large correlation was observed between pace and elevation accumulated (r = 0.677). We concluded that significant workload differences from one week to the next only occurred in preparation for longer-distance competitions, with sudden acute load decreases and very low ACWR values reported mainly in weeks 1 and 2 of the taper. Meaningful relationships were found between performance (pace) and MAS for longer trails and between pace and MAS for ultra-trail competitions.

## 1. Introduction

Quantifying load contributes to a more precise and individualized training process [1]. It also assists in pinpointing the impacts of exercise on organic and physical functions and maintaining balance in the fitness–fatigue relationship [2]. For these reasons, closely monitoring training load in preparation for competition is common across various modalities [3]. The training load has two components: the external load (the physical and mechanical dimension of the imposed exercise) and the internal load (the biological response to the exercise) [4]. Monitoring training load during training can optimize the sports planning process and ultimately improve an athlete’s performance [5]. Different instruments can be used in the implementation of training load monitoring; for example, the global satellite navigation system (GSNS) facilitates external load monitoring by analyzing the distances traveled by an athlete at different speed ranges [6]. Meanwhile, cardio-frequencimeters, which help to control heart rate, are often used in internal load monitoring. Effort scales—which are notable for their validity and reliability [7,8]—may also be used to measure rate of perceived exertion (RPE) as an element of internal load; the RPE scale is multiplied by session time (in minutes) to obtain a global indicator of the designated session-RPE load (s-RPE) [8].

Endurance sports constitute an extensive individual modality (duration and distance), causing numerous physiological changes to occur in the athlete [9]; thus, load monitoring can be crucial to performance improvement [2]. In a study developed on trail running, which employed load monitoring, the RPE values presented in male trail runners for the session are 213.7 ± 223.95 A.U. [10]. These training session values make it possible to quantify the weekly workload and to extrapolate that those weeks with values above 1500 A.U. are associated with an increased occurrence of injury and conditioning performance [11]. Spikes in load must be avoided; the acute:chronic workload ratio (ACWR) makes it possible to assess the chronic load and ensure that it is sufficient for the acute load being imposed on the athlete [12]. In this way, spikes in load create new stimuli and increase the athlete’s physical levels and, consequently, their performance [13]. Bearing in mind that the spikes originate variations in the load, they must be weighed across multiple weeks and within individual weeks to allow for appropriate load distribution based on the adaptation and effects on the athlete [14]. Other indices that can be calculated based on external or internal load indicators are the training monotony and training strain—both originally proposed by Foster [15], the training monotony being calculated through the workload’s average divided by the standard deviation of the workload and the training strain by monotony multiplied by the workload [15]. Using these two indices (monotony and strain), Matos et al. (2020) developed a study of trail running athletes that demonstrated that monotony values between 0.6 and 0.9 resulted in limitations in the athletes’ performance [16].

The primary goal of athletes and coaches being to improve performance in competition, the way to achieve this is through overcompensation caused by load [17]. For this effect to succeed, the training must be planned with great precision to involve phases of overload (e.g., high volumes, great intensity, and diversity of exercises) [18]. A tapering phase—marked by a reduction in load volume, but with sustained frequency and intensity—is used in many sports to incorporate more specific exercises [17,19]. In the tapering phase, athletes and coaches must pay special attention to the balance between the training volume and the length of the phase to ensure that the best performance coincides with the competition’s time [20]. In endurance sports, the meta-analysis developed by Bosquet et al. (2007) recommends a two-week tapering phase, involving volume reductions of 41%–60% while training frequency and intensity remain unchanged [21].

In any sport, workload indices are vital to understanding an athlete’s adaptations, through an appropriate training prescription, ensuring the best performance in competition [2]. Analyzing the variation in the athlete’s response to training is equally essential; the relationship between training and performance is a system of input and output, in which the athlete receives training (input) and generates a final competition performance (output) [22]. In this sense, the relationship can be likened to a dose-response effect in which the athlete’s physiological response is derived from their training load—the stimulus [23]. Using the dose-response relationship—which is modeled as an inverted U-curve—in the planning of an athlete’s training, it is possible to adjust the load to target the best performance [24].

Although the load is an essential variable to improve performance, there are intrinsic factors in the athlete that inevitably play an important role. For example, in modalities of extensive character, the maximum volume of oxygen appears to assume a considerable preponderance [25]. In this sense, the literature presents reliable tests such as the Cooper 12-min run test or 5-min field test, making it possible to estimate the maximum oxygen uptake and calculate the aerobic performance [26,27]. Therefore, through the interaction between an athlete’s training load and their physical capacity, in trail running also, it is essential to understand this, helping coaches and athletes to predict and plan effectively and contributing to the performance in competition.

However, to the authors’ knowledge, no study has been developed on the interaction of training load with performance in competition in trail running athletes. Furthermore, no research has been developed on training load interaction with competition over a trail running season. Thus, the objectives of the present study were (i) to analyze pace and workload indices in the four weeks leading up to different types of competitions, and (ii) to identify correlations between pace, workload indices, and other variables of physical fitness in different types of competitions (short trail (<21 km); long trail (22–42 km); ultra-trail medium (43–69 km); ultra-trail long/extra-long (>70 km).

## 2. Materials and Methods

### 2.1. Participants

Twenty-five recreational male trail running athletes participated in the study (age: 36.23 ± 8.30 years old; height: 172.12 ± 5.12 cm; body mass: 67.24 ± 5.97 kg; minimum International Trail Running Association performance index: 600). All were required to participate in the trail running championship (short trail (<21 km), long trail (22–42 km), ultra-trail medium (43–69 km), ultra-trail long/extra-long (>70 km)) in Portugal in the 2018/2019 season. Inclusion criteria included (i) participation in the national trail running championships, (ii) more than three years’ experience in the sport, (iii) registration in all training sessions, (iv) registration in all competitions, and (v) not having been injured for more than three consecutive weeks in the 12 months prior (aiming to allow determination of the chronic load for the period). Before the study began, all athletes were informed of the objectives, procedures, and protocol of the study and voluntarily signed an informed consent form. The study was carried out following the Helsinki Declaration’s (1964) ethics recommendations for studies on humans.

### 2.2. Experimental Approach to the Problem

**Figure 1:**
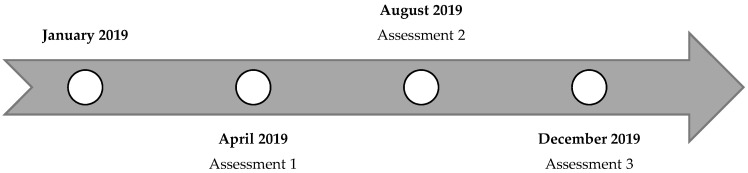
Timeline of assessments during 52 weeks of study.

This study followed a cohort study design. Over 52 weeks, all training sessions were monitored using global positioning systems (GPS) that quantified both the total distance covered and, with the use of the Borg CR-10 scale, the RPE and session-RPE. The athletes and their coaches determined the nature and content of the training sessions. A total of 148.12 ± 57.53 training sessions was analyzed for each athlete. Every four months (Figure 1), athletes were assessed for their anthropometry and aerobic performance; for each week of training, load indicators were used to calculate the acute load (the sum of the weekly training loads), the acute:chronic workload ratio, the training monotony, and the training strain. Training weeks were defined as starting on Monday and ending on Sunday. Throughout the testing, pace was calculated and used as the primary performance outcome.

Per the study’s objectives, training load indexes were analyzed and compared in the four weeks leading up to the competition. The weeks were classified as (i) competition week (the week during which the competition occurred), (ii) week −1 (the week of training before the competition), (iii) week −2 (two weeks before the competition), and (iv) week −3 (three weeks before the competition). Meanwhile, the trail running competitions were classified according to the Associação Trail Running Portugal, the organization responsible for trail running in Portugal as ST: short trail (<21 km), LT: long trail (22–42 km); UT-M: ultra-trail medium (43–69 km), and UT-L/XL: ultra-trail long/extra-long (>70 km).

### 2.3. Periodic Assessment

#### 2.3.1. Anthropometrics Composition

For bodyweight, we used the Tanita BC-601 (Tokyo, Japan, measured to the nearest 0.1 kg), and for height, the stadiometer Seca 217 (Hamburg, Germany, measured to the nearest 0.1 cm).

#### 2.3.2. Aerobic Performance

To assess aerobic performance, we asked all athletes to participate in a 5-min field test (with high validity and reliability), as described by Berthon et al. [27]. Before the test, the athletes performed a standard 5-min warm-up consisting of light running and lower limb mobility. The test was administered on a flat track during morning hours, with temperatures varying from 15 to 25 °C (depending on the hour and day). The athletes were instructed to maintain a constant pace and to avoid resting for the duration of the test to achieve maximal performance and recovery. Their total distance was recorded at the end of the 5 min. Their maximal aerobic speed (MAS) was determined by dividing the total distance in meters by the time in seconds, with the final result expressed in m/s (MAS = total distance/time).

### 2.4. Training Load Monitoring

#### 2.4.1. Distance Covered

Using the integrated GPS technology in the Polar V800 watch (37 mm × 56 mm × 12.7 mm, weight: 79 g) (Polar, Finland), athletes self-reported the total distance they covered in each of their training sessions. The selected watch model was tested for validity in previous studies and demonstrated acceptable values of accuracy [28].

#### 2.4.2. Rate of Perceived Exertion

Thirty minutes after the training session, athletes were asked the question, “How hard was the training session?” Their response—their RPE—was recorded using the Borg CR-10 scale [29]; this scale was introduced to the athletes two weeks before the study to ensure their familiarity and ability to provide precise responses. Based on the reported RPE value and the duration of the training session in minutes, the session-RPE was calculated and expressed in arbitrary units (A.U.) [6]. This process was repeated for all training sessions to quantify internal load [30].

#### 2.4.3. Workload Indices

Based on the variables of distance, duration, and sRPE, the following indices were determined: (i) weekly training load (the sum of all training loads for the week), (ii) acute:chronic workload ratio (ACWR: calculated by dividing the acute load (the current week’s training load) by the chronic workload (the average of the previous four weeks’ workloads)) [12], (iii) training monotony (the average of the last seven days’ workloads divided by the standard deviation of the last seven days’ workloads) [15], and (iv) training strain (monotony multiplied by workload) [15].

### 2.5. Competition Monitoring

#### Pace

From the races completed by the athletes and using the different categories, the time of the race and the distance covered were collected, calculating the pace.

### 2.6. Statistical Analysis

The results were expressed as means and standard deviations. The data were checked for normality (*p* > 0.05) and homogeneity (*p* > 0.05), and an ANOVA of repeated measures was subsequently performed to compare the workload indices (acute load, ACWR, training monotony, and training strain) of the variables (sRPE, total distance, and total time) between the four weeks—that is, the three weeks leading up to the competition and the week of the competition. Bonferroni’s post hoc test was used to analyze pairwise variations (week vs. week analysis). The level of statistical significance was set at *p* < 0.05. Additionally, the standardized effect size (ES) of Cohen’s d was calculated for pairwise comparisons. The magnitude of the ES was categorized based on the following thresholds: ≤0.2 (trivial), from 0.3 to 0.6 (small), from 0.6 to 1.2 (moderate), from 1.2 to 2.0 (large), and >2.0 (very large) [31]. The associations between pace, physical fitness, and workload indices were made with the Pearson correlation test (r), using the mean values of the four weeks leading up to the competition. The magnitude of the correlation was categorized based on the following thresholds: <0.1 (trivial), from 0.1 to 0.3 (small), from 0.3 to 0.5 (moderate), from 0.5 to 0.7 (large), from 0.7 to 0.9 (very large), and ≥0.9 (nearly perfect). SPSS Statistics software (version 24, IBM Corporation, Armonk, NY, USA) was used for the analysis.

## 3. Results

The pace in different categories of trail competitions can be found in Table 1, seven athletes having participated in the ST category, seven athletes in LT, six athletes in UT-M, and five athletes in UT-L/XL. The pace (in minutes per kilometer) for short trail (ST) was 6.36 ± 1.97; long trail (LT) was 6.83 ± 1.72; ultra-trail medium was 7.32 ± 1.41; and ultra-trail long or ultra-trail extra-long was 8.48 ± 1.57.

Table 2 presents the weekly variations of acute load, acute chronic workload ratio, training monotony and strain of total distance, total time and RPE before the ST competitions. No significant differences (*p* > 0.05) were found between the workload variables before the ST competitions. It was found that in ST competitions, the lowest acute load TD (36.28 km), and sRPE (752.93 A.U.) occurred in the week of competition, and the lowest acute load TT (202.14 min) occurred in week-2, while the greatest occurred in week-1, namely 42.34 km, 254.58 min and in week-3, that is 946.19 A.U. This represents a difference of −14.3% total distance from the week-1 to the week of competition, 25.9% of total time from the week-2 to the week-1, and −20.4% of sRPE from the week-3 to the week of competition.

Table 3 presents the weekly variations of acute load, acute chronic workload ratio, training monotony and strain of total distance, total time and RPE before the LT competitions. It was found that in LT competitions, the lowest acute load TD (34.19 km), TT (166.43 min) and sRPE (673.36 A.U.) occurred in the week of competition, while the greatest occurred in week-1, namely 53.93 km, 301.48 min and 1284.31 A.U. This represents a difference of −36.6% total distance, −44.8% of total time and −47.6% of sRPE from the week-1 to the week of competition.

Table 4 presents the weekly variations of acute load, acute chronic workload ratio, training monotony and strain of total distance, total time and RPE before the UT-M competitions. It was found that in UT-M competitions, the lowest acute load TD (26.23 km), TT (139.98 min) and sRPE (457.08 A.U.) occurred in the week of competition, while the greatest occurred in week-2, namely 56.78 km, 344.14 min and in week-1, that is 1183.43 A.U. This represents a difference of −53.8% total distance, −59.3% of total time to week-2 to the week of competition, and −61.4% of sRPE from the week-1 to the week of competition.

Table 5 presents the weekly variations of acute load, acute chronic workload ratio, training monotony and strain of total distance, total time and RPE before the UT-L/XL competitions. It was found that in UT-L/XL competitions, the lowest acute load TD (19.95 km), TT (113.81 min) and sRPE (326.23 A.U.) occurred in the week of competition, while the greatest occurred in week-3, namely 59.29 km, 355.48 min and 1447.33 A.U. This represents a difference of −66.4% total distance, −68% of total time and −77.5% of sRPE from the week-3 to the week of competition.

Table 6 presents the correlations between the pace and physical fitness and workload indices in different categories of trail competitions. Moderate correlations were found between the pace in ST with acute:chronic workload ratio of total distance (r = −0.334, 95% confidence interval-CI (−0.58; −0.04)), and pace in ST with training monotony of RPE (r = −0.303, 95% CI (− 0.55; 0)); while, pace in LT had moderate correlation with elevation accumulated (r = 0.425, 95% CI (0.24;0.58)). Concerning the pace in UT-M, small correlations were found with acute load of RPE (r = −0.298, 95% CI(−0.51; −0.05)), acute:chronic workload ratio of total distance (r = −0.253, 95%CI (−0.47; −0.01)), acute:chronic workload ratio of RPE (r = −0.263, 95% CI (−0.48; −0.02)), moderate correlations were found with maximal aerobic speed (r = −0.398, 95% CI (−0.59; −0.17)), training strain of total distance (r = −0.305, 95% CI (−0.51; −0.06)), total time (r = −0.305, 95% CI (−0.51; −0.06)), RPE (r = −0.305, 95% CI (−0.51; −0.06)), and very large correlations were found with elevation accumulated (r = 0.703, 95% CI (0.55; 0.81)). In relation to pace in UT-L/XL large correlation were found with elevation accumulated (r = 0.677, 95% CI (0.42; 0.83)).

## 4. Discussion

The purpose of this study was to analyze training load variations within the four weeks before the competition, as well as the associations between pace, physical fitness, and workload indices for the four running competitions. The main finding was that all competitions except the ST saw significant workload differences in the preceding weeks. Large to very large correlations were found between pace and ELac for UT-M and UT-L/XL competitions, while only UT-M showed associations between pace and physical fitness.

Despite a lack of meaningful differences in workload measures during the weeks leading up to the ST competitions, it was found that the workload indices of the overall measures decreased from week-3 to week-2. From week-2 to week-1, the workload indices increased again; then, they tapered off the week before the competition. The acute load decreases and increases for TD, TT, and sRPE varied from −16% to 26%. Given the considerable variation in workloads, it is important to consider the “10 percent rule” in the association between workload and performance, since when variations between weeks greater than 10% are verified, this can condition performance [32].

The ACWR, TM, and TS of TD, TT, and sRPE values remained in the safe zones in the weeks before competition. Although the TM values reported in the present study are considered “safe”, as the “danger” threshold is considered to be at approximately 2.0 A.U. [15], in a study conducted on 25 trail running athletes to analyze their workload indices in the three weeks preceding an injury, it was found that TM values remained consistently low until the last week [16]. These data should be analyzed with some caution, as small increases (<10%) to weekly training load in athletes with low chronic loads may result in a low dose-response to training, while the same increase for athletes with extremely high chronic loads may be too much [33]. Determining whether the ACWR thresholds of other sport contexts are comparable to those expected in trail running may also provide useful insights [11].

Longer distance competitions (LT and UT-M) saw a similar pattern of progressive increase in overall acute loads and workload indices from week-3 to week-2, followed by a 2-week tapering phase that resulted in a decrease of up to −53.8% and −59.3% for TD and TT, respectively. Meanwhile, the extra-long competition (UT-L/XL) saw a “wave-like” workload pattern, similar to the ST competition. The longer the trail running competition, the greater the decrease in load from week-1 of training to the week of the competition (−60%, −64%, and −75% for TD, TT, and sRPE, respectively). There remains no consensus as to which tapering technique is best (i.e., the “optimal duration”), especially when it comes to longer distance competitions [20].

Notwithstanding the “optimal” recovery strategy issue, some authors recommend training volume reductions of 40–80% [20,34]. However, massive reductions in training volume and frequency can also increase a runner’s risk of detraining and ultimately reduce their performance [35]. It is worth noting that the data in the present study included significant variation. The standard deviations of the overall workload indices remained high (some even greater than the mean) for all competitions; this indicates that within-subject variation was high and was increasingly accentuated in longer competitions. Also noteworthy were the ACWR values for TD, TT, and sRPE, which dropped well below 0.8 A.U. in the last week of taper (competition week)—a potential danger zone for detraining [13].

Regarding the second objective of the present study, it was found that pacing increased with competition distance. The pace in longer competitions showed large and very large correlations with elevation accumulated (Elac); the longer the competition, the greater the Elac, as more uphill terrain is incorporated into the course. It is possible that athletes feel added pressure and motivation to make up time during such courses, given that their pacing is reduced during hill climbs [36,37]. In fact, this pattern was observed in a study conducted on 50 trail runners competing in trail ultramarathons over a period of two years. The runners’ mean pace was 9.23 ± 1.13 min/km—which is similar to the UT-L/XL competition pacing in the present study [38]—and, indeed, the data showed that the athletes slowed their pace (to between 8 and 10 min/km) during longer climbs, and then regained their pace (up to 14 min/km) on downhills and shorter climbs with lower altitudes [38]. Only moderate correlations were found between pace and Elac for UT-M competitions. Based on this data, a similar study conducted on 23 recreational trail runners in a 65 km competition showed a moderate correlation between VO2max and performance; the authors associated higher values of VO2max with the submaximal intensities observed in competitions of longer duration [25]. Coaches should assess and monitor VO2max and MAS values during the training process, as this information may influence performance even in longer distance competitions [39].

The present study showed that the longer the trail running competition, the more the acute loads for TD, TT, and sRPE will decrease in the week before a competition. The shortest (ST) and the longest (UT-L/XL) trail competitions each used a one-week taper, while the LT and UT-M competitions used a two-week taper—revealing large decreases in ACWR values for TD, TT, and sRPE variables in all competitions. Pace showed a large to very large correlation with Elac in longer distance competitions, but only the UT-M competition showed a moderate correlation between pace and MAS.

This study also had its limitations, one of which is quantifying metabolic demands as a function of workload, suggesting that the intake of 120 g/h of carbohydrates may limit metabolic fatigue and exercise-induced muscle damage during ultra-endurance races [40,41]. In terms of the sample size, only male athletes were included. Including female athletes in future studies of this nature would be pertinent, as trail running competitions are seeing an increasing number of female participants. Another limitation was the experience level of the sample; as non-professionals, the athletes were not necessarily accustomed to using sRPE to rate their efforts. Incorporating other GPS metrics into such a study—such as accelerations, decelerations, and impacts—would also offer interesting insights regarding non-linear courses and how they may impact an athlete’s risk of injury. Information on carbohydrate intake should also be a variable to include in future studies, which may cause differences in loads’ perception. Additionally, collecting data on elevation accumulated—including intra-week analyses of uphill cumulative load during training—would be useful for future studies.

## 5. Conclusions

In our analysis of trail running competitions, pace was found to increase with the length of the competition. Only the longer distance competitions (from LT to UT-L/XL) showed significant workload differences between weeks, with sudden acute load decreases and very low ACWR values for TD, TT, and sRPE primarily in the one–two weeks of taper. Meaningful correlations were observed between performance (pace) and Elac for longer trails, and between pace and MAS for UT-M competitions only. These results highlight the impact of taper strategies when preparing for a competition and navigating the potential risk of detraining. Exposure to the cumulative elevations encountered in a competition is also an important aspect of an athlete’s training, particularly when preparing for a longer distance trail running competition.

Our results suggest that coaches and athletes should pay special attention to variations in workload before the competition and the tapering effect based on the race category, thus enhancing the best performance at the right time (race day). On the other hand, and regarding pace, through this study it becomes possible to understand which indices support each category of race, offering an essential tool in preparing trail running races.

## Figures and Tables

**Table 1:** Descriptive statistics of pace in different categories of trail competitions.

	ST	LT	UT-M	UT-L/XL
Pace in competition (min/km)	6.36 ± 1.97	6.83 ± 1.72	7.32 ± 1.41	8.48 ± 1.57

ST: short trail (<21 km); LT: long trail (22–42 km); UT-M: ultra-trail medium (43–69 km); UT-L/XL: ultra-trail long/extra-long (>70 km).

**Table 2:** Descriptive statistics of workload measures before short trail (ST) competitions.

	wC	w-1	w-2	w-3	*p*
alTD (km)	36.28 ± 26.28	42.34 ± 33.57	35.97 ± 25.65	42.08 ± 29.67	0.466
alTT (min)	202.50 ± 139.13	254.58 ± 226.67	202.14 ± 145.65	241.53 ± 189.55	0.344
alsRPE (A.U.)	752.93 ± 514.97	877.02 ± 822.97	791.49 ± 633.43	946.19 ± 926.34	0.816
acwrTD (A.U.)	0.95 ± 0.57	0.93 ± 0.66	0.88 ± 0.51	0.96 ± 0.57	0.962
acwrTT (A.U.)	0.95 ± 0.59	0.87 ± 0.62	0.86 ± 0.52	0.96 ± 0.62	0.784
acwrRPE (A.U.)	0.95 ± 0.66	0.97 ± 0.83	0.88 ± 0.63	0.97 ± 0.73	0.940
tmTD (A.U.)	0.66 ± 0.35	0.69 ± 0.41	0.71 ± 0.44	0.75 ± 0.39	0.601
tmTT (A.U.)	0.63 ± 0.31	0.65 ± 0.38	0.68 ± 0.42	0.71 ± 0.35	0.582
tmRPE (A.U.)	0.60 ± 0.29	0.62 ± 0.38	0.63 ± 0.35	0.67 ± 0.31	0.673
tsTD (A.U.)	24.24 ± 20.23	37.38 ± 41.68	35.13 ± 43.72	40.67 ± 40.11	0.203
tsTT (A.U.)	122.00 ± 86.70	206.21 ± 243.19	181.46 ± 213.21	213.02 ± 211.19	0.229
tsRPE (A.U.)	462.59 ± 355.67	787.62 ± 1009.31	531.65 ± 465.86	824.47 ± 933.34	0.207

wC: week of the competition; w-1: one week before the competition; w-2: two weeks before the competition; w-3: three weeks before the competition; alTD: acute load total distance; alTT: acute load total time; alsRPE: acute load session-RPE; acwrTD: acute:chronic workload ratio total distance; acwrTT: acute:chronic workload ratio total time; acwrRPE: acute:chronic workload ratio RPE; tmTD: training monotony total distance; tmTT: training monotony total time; tmRPE: training monotony RPE; tsTD: training strain total distance; tsTT: training strain total time; tsRPE: training strain RPE; ES: effect size.

**Table 3:** Descriptive statistics of workload measures before long trail (LT) competitions.

	wC	Week-1	Week-2	Week-3	*p*	Post Hoc	ES
alTD (km)	34.19 ± 26.00	53.93 ± 32.81	48.17 ± 28.54	45.84 ± 31.14	≤0.001	wC vs. w-1: ≤0.001 * wC vs. w-2: ≤0.001 * wC vs. w-3: 0.014 *	wC vs. w-1: −0.658 ^$^ wC vs. w-2: −0.491 ^@^ wC vs. w-3: −0.392 ^@^
alTT (min)	166.43 ± 119.07	301.48 ± 188.15	260.06 ± 173.73	267.01 ± 184.84	≤0.001	wC vs. w-1: ≤0.001 * wC vs. w-2: ≤0.001 * wC vs. w-3: ≤0.001 *	wC vs. w-1: −0.842 ^$^ wC vs. w-2: −0.613 ^$^ wC vs. w-3: −0.628 ^$^
alsRPE (A.U.)	673.36 ± 631.35	1284.31 ± 1081.89	1127.22 ± 992.69	1085.70 ± 974.01	≤0.001	wC vs. w-1: ≤0.001 * wC vs. w-2: ≤0.001 * wC vs. w-3: 0.003 *	wC vs. w-1: −0.674 ^$^ wC vs. w-2: −0.550 ^@^ wC vs. w-3: −0.490 ^@^
acwrTD (A.U.)	0.73 ± 0.48	1.11 ± 0.57	1.04 ± 0.58	0.98 ± 0.67	≤0.001	wC vs. w-1: ≤0.001 * wC vs. w-2: ≤0.001 * wC vs. w-3: 0.045 *	wC vs. w-1: −0.721 ^$^ wC vs. w-2: −0.582 ^@^ wC vs. w-3: −0.429 ^@^
acwrTT (A.U.)	0.66 ± 0.41	1.09 ± 0.59	1.02 ± 0.62	0.99 ± 0.70	≤0.001	wC vs. w-1: ≤0.001 * wC vs. w-2: ≤0.001 * wC vs. w-3: 0.003 *	wC vs. w-1: −0.836 ^$^ wC vs. w-2: −0.712 ^$^ wC vs. w-3: −0.575 ^@^
acwrRPE (A.U.)	0.65 ± 0.46	1.10 ± 0.66	1.01 ± 0.64	1.00 ± 0.79	≤0.001	wC vs. w-1: ≤0.001 * wC vs. w-2: ≤0.001 * wC vs. w-3: 0.005 *	wC vs. w-1: −0.790 ^$^ wC vs. w-2: −0.671 ^$^ wC vs. w-3: −0.557 ^@^
tmTD (A.U.)	0.64 ± 0.34	0.91 ± 0.47	0.85 ± 0.42	0.77 ± 0.41	≤0.001	wC vs. w-1: ≤0.001 * wC vs. w-2: ≤0.001 *	wC vs. w-1: −0.636^$^ wC vs. w-2: −0.593 ^@^
tmTT (A.U.)	0.63 ± 0.33	0.87 ± 0.43	0.79 ± 0.36	0.75 ± 0.41	≤0.001	wC vs. w-1: ≤0.001 * wC vs. w-2: ≤0.001 *	wC vs. w-1: −0.627 ^$^ wC vs. w-2: −0.559 ^@^
tmRPE (A.U.)	0.59 ± 0.29	0.79 ± 0.35	0.73 ± 0.31	0.67 ± 0.33	≤0.001	wC vs. w-1: ≤0.001 * wC vs. w-2: ≤0.001 *	wC vs. w-1: −0.633^$^ wC vs. w-2: −0.552 ^@^
tsTD (A.U.)	21.74 ± 19.45	57.33 ± 52.00	49.23 ± 45.09	41.54 ± 40.37	≤0.001	wC vs. w-1: ≤0.001 * wC vs. w-2: ≤0.001 * wC vs. w-3: ≤0.001 *	wC vs. w-1: −0.855 ^$^ wC vs. w-2: −0.781 ^$^ wC vs. w-3: −0.579 ^@^
tsTT (A.U.)	115.06 ± 103.18	306.57 ± 270.99	249.09 ± 224.28	222.43 ± 213.18	≤0.001	wC vs. w-1: ≤0.001* wC vs. w-2: ≤0.001 * wC vs. w-3: ≤0.001 *	wC vs. w-1: −0.926 ^$^ wC vs. w-2: −0.813 ^$^ wC vs. w-3: −0.642 ^$^
tsRPE (A.U.)	404.46 ± 432.03	1033.02 ± 1003.36	971.43 ± 1007.67	839.89 ± 931.03	≤0.001	wC vs. w-1: ≤0.001 * wC vs. w-2: ≤0.001 * wC vs. w-3: 0.005 *	wC vs. w-1: −0.806 ^$^ wC vs. w-2: −0.675 ^$^ wC vs. w-3: −0.543 ^@^

wC: week of the competition; w-1: one week before the competition; w-2: two weeks before the competition; w-3: three weeks before the competition; alTD: acute load total distance; alTT: acute load total time; alsRPE: acute load session-RPE; acwrTD: acute:chronic workload ratio total distance; acwrTT: acute:chronic workload ratio total time; acwrRPE: acute:chronic workload ratio RPE; tmTD: training monotony total distance; tmTT: training monotony total time; tmRPE: training monotony RPE; tsTD: training strain total distance; tsTT: training strain total time; tsRPE: training strain RPE; ES: effect size; *: statistically significant at a *p* < 0.05; ES: effect size; ^@^: small ES; ^$^: moderate ES.

**Table 4:** Descriptive statistics of workload measures before ultra-trail medium (UT-M) competitions.

	wC	Week-1	Week-2	Week-3	*p*	Post Hoc	ES
alTD (km)	26.23 ± 21.18	53.79 ± 30.33	56.78 ± 38.87	42.01 ± 33.58	≤0.001	wC vs. w-1: ≤0.001 * wC vs. w-2: ≤0.001 * wC vs. w-3: 0.008 *	wC vs. w-1: −1.054 ^$^ wC vs. w-2: −0.976 ^$^ wC vs. w-3: −0.562 ^@^
alTT (min)	139.98 ± 115.65	328.19 ± 190.74	344.14 ± 253.12	248.00 ± 198.86	≤0.001	wC vs. w-1: ≤0.001 * wC vs. w-2: ≤0.001 * wC vs. w-3: 0.002 *	wC vs. w-1: −1.193 ^$^ wC vs. w-2: −1.038 ^$^ wC vs. w-3: −0.664 ^$^
alsRPE (A.U.)	457.08 ± 427.23	1183.43 ± 859.84	1142.84 ± 995.41	927.31 ± 909.41	≤0.001	wC vs. w-1: ≤0.001 * wC vs. w-2: ≤0.001 ^*^ wC vs. w-3: 0.002 *	wC vs. w-1: −1.158 ^$^ wC vs. w-2: −0.975 ^$^ wC vs. w-3: −0.764 ^$^
acwrTD (A.U.)	0.56 ± 0.44	1.07 ± 0.49	1.16 ± 0.64	1.05 ± 0.73	≤0.001	wC vs. w-1: ≤0.001 * wC vs. w-2: ≤0.001 * wC vs. w-3: ≤0.001 *	wC vs. w-1: −1.128 ^$^ wC vs. w-2: −1.119 ^$^ wC vs. w-3: −0.877 ^$^
acwrTT (A.U.)	0.52 ± 0.43	1.08 ± 0.51	1.20 ± 0.67	1.05 ± 0.76	≤0.001	wC vs. w-1: ≤0.001 * wC vs. w-2: ≤0.001 * wC vs. w-3: ≤0.001 *	wC vs. w-1: −1.220 ^#^ wC vs. w-2: −1.220 ^#^ wC vs. w-3: −0.921 ^$^
acwrRPE (A.U.)	0.44 ± 0.36	1.09 ± 0.67	1.21 ± 0.78	1.06 ± 0.85	≤0.001	wC vs. w-1: ≤0.001 * wC vs. w-2: ≤0.001 * wC vs. w-3: ≤0.001 *	wC vs. w-1: −1.209 ^#^ wC vs. w-2: −1.301 ^#^ wC vs. w-3: −0.965 ^$^
tmTD (A.U.)	0.57 ± 0.33	0.83 ± 0.41	0.86 ± 0.46	0.73 ± 0.45	≤0.001	wC vs. w-1: ≤0.001 * wC vs. w-2: ≤0.001 * wC vs. w-3: 0.016 *	wC vs. w-1: −0.831 ^$^ wC vs. w-2: −0.818 ^$^ wC vs. w-3: −0.520 ^@^
tmTT (A.U.)	0.56 ± 0.31	0.78 ± 0.33	0.82 ± 0.43	0.67 ± 0.39	≤0.001	wC vs. w-1: ≤0.001 * wC vs. w-2: ≤0.001 *	wC vs. w-1: −0.817 ^$^ wC vs. w-2: −0.769 ^$^
tmRPE (A.U.)	0.53 ± 0.28	0.75 ± 0.33	0.78 ± 0.40	0.65 ± 0.37	≤0.001	wC vs. w-1: ≤0.001 * wC vs. w-2: ≤0.001 * wC vs. w-3: 0.048 *	wC vs. w-1: −0.820 ^$^ wC vs. w-2: −0.777 ^$^ wC vs. w-3: −0.435 ^@^
tsTD (A.U.)	17.53 ± 18.43	51.45 ± 47.81	53.97 ± 50.63	38.19 ± 41.91	≤0.001	wC vs. w-1: ≤0.001 * wC vs. w-2: ≤0.001 * wC vs. w-3: 0.003 *	wC vs. w-1: −0.965 ^$^ wC vs. w-2: −0.930 ^$^ wC vs. w-3: −0.669 ^$^
tsTT (A.U.)	90.92 ± 94.59	284.57 ± 245.93	300.18 ± 259.90	208.25 ± 218.37	≤0.001	wC vs. w-1: ≤0.001 * wC vs. w-2: ≤0.001 * wC vs. w-3: ≤0.001 *	wC vs. w-1: −1.120 ^$^ wC vs. w-2: −1.045 ^$^ wC vs. w-3: −0.730 ^$^
tsRPE (A.U.)	282.70 ± 315.00	958.68 ± 907.91	949.92 ± 903.27	740.34 ± 849.62	≤0.001	wC vs. w-1: ≤0.001 * wC vs. w-2: ≤0.001 * wC vs. w-3: ≤0.001 *	wC vs. w-1: −1.036 ^$^ wC vs. w-2: −1.066 ^$^ wC vs. w-3: −0.817 ^$^

wC: week of the competition; w-1: one week before the competition; w-2: two weeks before the competition; w-3: three weeks before the competition; alTD: acute load total distance; alTT: acute load total time; alsRPE: acute load session-RPE; acwrTD: acute:chronic workload ratio total distance; acwrTT: acute:chronic workload ratio total time; acwrRPE: acute:chronic workload ratio RPE; tmTD: training monotony total distance; tmTT: training monotony total time; tmRPE: training monotony RPE; tsTD: training strain total distance; tsTT: training strain total time; tsRPE: training strain RPE; ES: effect size; *: statistically significant at a *p* < 0.05; ES: effect size; ^@^: small ES; ^$^: moderate ES; ^#^: large ES.

**Table 5:** Descriptive statistics of workload measures before ultra-trail long/extra-long (UT-L/XL) competitions.

	wC	Week-1	Week-2	Week-3	*p*	Post Hoc	ES
alTD (km)	19.95 ± 12.60	50.00 ± 28.75	50.81 ± 33.68	59.29 ± 39.80	≤0.001	wC vs. w-1: ≤0.001 * wC vs. w-2: ≤0.001 * wC vs. w-3: ≤0.001 *	wC vs. w-1: −1.354 ^#^ wC vs. w-2: −1.213 ^#^ wC vs. w-3: −1.333 ^#^
alTT (min)	113.81 ± 70.79	313.97 ± 185.75	329.61 ± 243.79	355.48 ± 250.85	≤0.001	wC vs. w-1: ≤0.001 * wC vs. w-2: ≤0.001 * wC vs. w-3: ≤0.001 *	wC vs. w-1: −1.424 ^#^ wC vs. w-2: −1.202 ^#^ wC vs. w-3: −1.311 ^#^
alsRPE (A.U.)	326.23 ± 260.28	1280.55 ± 876.03	1157.14 ± 945.79	1447.33 ± 1262.24	≤0.001	wC vs. w-1: ≤0.001 * wC vs. w-2: ≤0.001 * wC vs. w-3: ≤0.001 *	wC vs. w-1: −1.443 ^#^ wC vs. w-2: −1.195 ^$^ wC vs. w-3: −1.256 ^#^
acwrTD (A.U.)	0.51 ± 0.44	0.88 ± 0.47	0.94 ± 0.55	1.09 ± 0.53	≤0.001	wC vs. w-1: 0.008 * wC vs. w-2: 0.005 * wC vs. w-3: ≤0.001 *	wC vs. w-1: −0.835 ^$^ wC vs. w-2: −0.965 ^$^ wC vs. w-3: −1.218 ^#^
acwrTT (A.U.)	0.49 ± 0.44	0.90 ± 0.47	0.91 ± 0.54	1.07 ± 0.56	≤0.001	wC vs. w-1: 0.007 * wC vs. w-2: 0.009 * wC vs. w-3: 0.002 *	wC vs. w-1: −0.891 ^$^ wC vs. w-2: −0.915 ^$^ wC vs. w-3: −1.130 ^$^
acwrRPE (A.U.)	0.41 ± 0.46	0.98 ± 0.70	0.89 ± 0.63	1.04 ± 0.54	≤0.001	wC vs. w-1: ≤0.001 * wC vs. w-2: 0.020 * wC vs. w-3: ≤0.001 *	wC vs. w-1: −0.962 ^$^ wC vs. w-2: −0.870 ^$^ wC vs. w-3: −1.256 ^#^
tmTD (A.U.)	0.49 ± 0.24	0.77 ± 0.45	0.70 ± 0.33	0.77 ± 0.44	0.002	wC vs. w-1: 0.002 * wC vs. w-2: 0.028 * wC vs. w-3: 0.003 *	wC vs. w-1: −0.744 ^$^ wC vs. w-2: −0.675 ^$^ wC vs. w-3: −0.810 ^$^
tmTT (A.U.)	0.49 ± 0.24	0.73 ± 0.41	0.67 ± 0.31	0.72 ± 0.40	0.004	wC vs. w-1: 0.004 * wC vs. w-3: 0.005 *	wC vs. w-1: −0.666 ^$^ wC vs. w-3: −0.720 ^$^
tmRPE (A.U.)	0.47 ± 0.23	0.70 ± 0.42	0.63 ± 0.29	0.72 ± 0.41	0.006	wC vs. w-1: 0.005 * wC vs. w-2: 0.043 * wC vs. w-3: 0.008 *	wC vs. w-1: −0.679 ^$^ wC vs. w-2: −0.611 ^$^ wC vs. w-3: −0.752 ^$^
tsTD (A.U.)	12.15 ± 10.39	49.15 ± 43.69	44.09 ± 39.35	49.88 ± 51.18	≤0.001	wC vs. w-1: ≤0.001 * wC vs. w-2: 0.002 * wC vs. w-3: 0.003 *	wC vs. w-1: −1.086 ^$^ wC vs. w-2: −1.134 ^$^ wC vs. w-3: −1.031 ^$^
tsTT (A.U.)	67.29 ± 53.84	284.10 ± 253.87	269.68 ± 244.20	269.97 ± 268.55	≤0.001	wC vs. w-1: ≤0.001 * wC vs. w-2: 0.002 * wC vs. w-3: 0.002 *	wC vs. w-1: −1.095 ^$^ wC vs. w-2: −1.152 ^$^ wC vs. w-3: −1.052 ^$^
tsRPE (A.U.)	183.79 ± 161.78	1033.11 ± 881.36	958.90 ± 985.45	695.25 ± 581.55	≤0.001	wC vs. w-1: ≤0.001 * wC vs. w-2: 0.010 * wC vs. w-3: ≤0.001 *	wC vs. w-1: −1.227 ^#^ wC vs. w-2: −1.021 ^$^ wC vs. w-3: −1.275 ^#^

wC: week of the competition; w-1: one week before the competition; w-2: two weeks before the competition; w-3: three weeks before the competition; alTD: acute load total distance; alTT: acute load total time; alsRPE: acute load session-RPE; acwrTD: acute:chronic workload ratio total distance; acwrTT: acute:chronic workload ratio total time; acwrRPE: acute:chronic workload ratio RPE; tmTD: training monotony total distance; tmTT: training monotony total time; tmRPE: training monotony RPE; tsTD: training strain total distance; tsTT: training strain total time; tsRPE: training strain RPE; ES: effect size; *: statistically significant at a *p* < 0.05; ES: effect size; ^$^: moderate ES; ^#^: large ES.

**Table 6:** Correlation coefficients between pace and physical fitness and workload indices.

	Pace in ST	Pace in LT	Pace in UT-M	Pace in UT-L/XL
ELac (mt)	r = 0.177 (−0.13; 0.45) ^@^	r = 0.425 (0.24; 0.58) **^,$^	r = 0.703 (0.55; 0.81) **^,~^	r = 0.677 (0.42; 0.83) **^,#^
MAS (m/s)	r = −0.027 (−0.32; 0.28) ^&^	r = 0.198 (−0.01; 0.39) ^@^	r = −0.398 (−0.59; −0.17) **^,$^	r = −0.229 (−0.54; 0.14) ^@^
alTD (km)	r = −0.293 (−0.55; 0.01) ^@^	r = 0.058 (−0.15; 0.26) ^&^	r = −0.233 (−0.45; 0.02) ^@^	r = 0.014 (−0.34; 0.37) ^&^
alTT (min)	r = −0.293 (−0.55; 0.01) ^@^	r = 0.042 (−0.17; 0.25) ^&^	r = −0.244 (−0.46; 0) ^@^	r = 0.082 (−0.28; 0.42) ^&^
alsRPE (A.U.)	r = −0.166 (−0.44; 0.14) ^@^	r = 0.035 (−0.17; 0.24) ^&^	r = −0.298 (−0.51; −0.05) *^, @^	r = 0.067 (−0.29; 0.41) ^&^
acwrTD (A.U.)	r = −0.334 (−0.58; −0.04) *^,$^	r = −0.058 (−0.26; 0.15) ^&^	r = −0.253 (−0.47; −0.01) *^,@^	r = −0.004 (−0.36; 0.35) ^&^
acwrTT (A.U.)	r = −0.299 (−0.55; 0) ^@^	r = −0.058 (−0.26; 0.15) ^&^	r = −0.237 (−0.46; 0.01) ^@^	r = −0.038 (−0.39; 0.32) ^&^
acwrRPE (A.U.)	r = −0.273 (−0.53; 0.03) ^@^	r = −0.064 (−0.27; 0.15) ^&^	r = −0.263 (−0.48; −0.02) *^,@^	r = −0.026 (−0.38; 0.33) ^&^
tmTD (A.U.)	r = −0.268 (−0.53; 0.04) ^@^	r = 0.064 (−0.15; 0.27) ^&^	r = −0.239 (−0.46; 0.01) ^@^	r = −0.062 (−0.41; 0.3) ^&^
tmTT (A.U.)	r = −0.260 (−0.52; 0.04) ^@^	r = 0.092 (−0.12; 0.29) ^&^	r = −0.224 (−0.45; 0.03) ^@^	r = −0.082 (−0.42; 0.28) ^&^
tmRPE (A.U.)	r = −0.303 (−0.55; 0) *^,$^	r = 0.075 (−0.14; 0.28) ^&^	r = −0.175 (−0.41; 0.08) ^@^	r = −0.053 (−0.4; 0.31) ^&^
tsTD (A.U.)	r = −0.271 (−0.53; 0.03) ^@^	r = −0.007 (−0.21; 0.2) ^&^	r = −0.305 (−0.51; −0.06) *^,$^	r = 0.011 (−0.34; 0.36) ^&^
tsTT (A.U.)	r = −0.269 (−0.53; 0.03) ^@^	r = −0.011 (−0.22; 0.2) ^&^	r = −0.305 (−0.51; −0.06) *^,$^	r = 0.040 (−0.32; 0.39) ^&^
tsRPE (A.U.)	r = −0.203 (−0.47; 0.1) ^@^	r = −0.004 (−0.21; 0.2) ^&^	r = −0.305 (−0.51; −0.06) *^,$^	r = 0.049 (−0.31; 0.40) ^&^

ELac: Elevation accumulated; MAS: Maximal aerobic speed; alTD: acute load total distance; alTT: acute load total time; alsRPE: acute load session-RPE; acwrTD: acute:chronic workload ratio total distance; acwrTT: acute:chronic workload ratio total time; acwrRPE: acute:chronic workload ratio RPE; tmTD: training monotony total distance; tmTT: training monotony total time; tmRPE: training monotony RPE; tsTD: training strain total distance; tsTT: training strain total time; tsRPE: training strain RPE; * Correlation is significant at *p* < 0.05; ** Correlation is significant at ≤0.01; ^&^: trivial magnitude; ^@^: small magnitude; ^$^: moderate magnitude; ^#^: large magnitude; ^~^: very large magnitude.
